# Temporal Monitoring of Differentiated Human Airway Epithelial Cells Using Microfluidics

**DOI:** 10.1371/journal.pone.0139872

**Published:** 2015-10-05

**Authors:** Cornelia Blume, Riccardo Reale, Marie Held, Timothy M. Millar, Jane E. Collins, Donna E. Davies, Hywel Morgan, Emily J. Swindle

**Affiliations:** 1 Academic Unit of Clinical and Experimental Sciences, Faculty of Medicine, University of Southampton, Southampton, United Kingdom; 2 Electronics and Computer Sciences, Faculty of Physical Sciences and Engineering, University of Southampton, Southampton, United Kingdom; 3 Institute for Life Sciences, University of Southampton, Southampton, United Kingdom; 4 National Institute for Health Research, Respiratory Biomedical Research Unit, University Hospital Southampton, Southampton, United Kingdom; University of Palermo, ITALY

## Abstract

The airway epithelium is exposed to a variety of harmful agents during breathing and appropriate cellular responses are essential to maintain tissue homeostasis. Recent evidence has highlighted the contribution of epithelial barrier dysfunction in the development of many chronic respiratory diseases. Despite intense research efforts, the responses of the airway barrier to environmental agents are not fully understood, mainly due to lack of suitable *in vitro* models that recapitulate the complex *in vivo* situation accurately. Using an interdisciplinary approach, we describe a novel dynamic 3D *in vitro* model of the airway epithelium, incorporating fully differentiated primary human airway epithelial cells at the air-liquid interface and a basolateral microfluidic supply of nutrients simulating the interstitial flow observed *in vivo*. Through combination of the microfluidic culture system with an automated fraction collector the kinetics of cellular responses by the airway epithelium to environmental agents can be analysed at the early phases for the first time and with much higher sensitivity compared to common static *in vitro* models. Following exposure of primary differentiated epithelial cells to pollen we show that CXCL8/IL–8 release is detectable within the first 2h and peaks at 4–6h under microfluidic conditions, a response which was not observed in conventional static culture conditions. Such a microfluidic culture model is likely to have utility for high resolution temporal profiling of toxicological and pharmacological responses of the airway epithelial barrier, as well as for studies of disease mechanisms.

## Introduction

The barrier functions of the airway epithelium are centrally involved in regulating tissue homeostasis in the lung and abnormal responses of the airway epithelium to the environment are thought to contribute to the pathogenesis of chronic airway diseases like asthma and chronic obstructive pulmonary disease (COPD) [1]. However, despite intensive research, there are still unmet medical needs in these diseases. One reason for this is the limited translation of data from animal models into the human situation due to differences in lung morphology, physiology and immunology [2]. Therefore, there is an urgent need for alternative human tissue or cell-based *in vitro* models in order to study the barrier functions of the airway epithelium as close as possible to the *in vivo* situation [3].

Most commonly used *in vitro* models of the airway epithelium consist of monolayers of cells growing on the surface of standard cell culture wells or more complex models in which cells are grown on a suspended porous support (e.g. Transwells^®^) to facilitate polarization and, under certain conditions, differentiation [3]. Even when cells are able to differentiate and recapitulate the *in vivo* structure, these systems are limited by their static nature. This is not representative of the *in vivo* situation where there is constant circulation of fluids that supply and remove an array of material (nutrients, metabolic waste products, hormones, mediators etc.) within the local tissue environment. Thus, the interstitial fluid flow contributes to tissue maintenance and homeostasis. Apart from its role in mass transport, interstitial fluid flow also provides a specific physiological microenvironment (including sheer forces, viscosity and local pressures) that is important for the normal functioning of interstitial cells such as fibroblasts and endothelial cells [4, 5]. Recently, a new class of cell culture devices, so-called organs-on-a-chip, have been developed incorporating continuous fluid flow for maintaining different cell types or tissue constructs such as liver, skin, kidney, gut, lung, heart and brain [6]. With this approach, long term cultures have been achieved and features that were not visible in static cultures have been highlighted. ‘Lung-on-a-chip’ models have been used to analyse the effects of flow rate [7], air liquid interface (ALI) culture under positive pressure [8], cyclic mechanical stresses [9, 10] and air plugs [11] have on cellular viability, phenotype and response to chemical stimuli/drugs. Organ-on-chip *in vitro* models mostly use pulmonary cell lines, which do not recapitulate the differentiated epithelium observed *in vivo*. The compatibility of these devices with fully differentiated primary human lung epithelial cells has still to be demonstrated. Additionally, it is preferable for lung *in vitro* models to include air-liquid interface culture in order to be as close as possible to the *in vivo* situation. Several approaches have used the convenience and robustness of commercially available permeable filter supports which allow cells to be grown in advance using established cell culture techniques and to maintain quality control of the cultures. For example, one organ-chip model has used a microfluidic circuit sustained by an integrated micro-pump to create a multi-organ-chip comprised of different cell types grown in filter supports to examine cross talk between two tissue types (e.g. skin and liver) [12, 13]. Other models have used a microfluidic channel to apply airflow to the apical side of differentiated primary bronchial epithelial cells (PBECs) in order to analyse the effect of sheer stress on ciliary function [14, 15]. However, none of the current devices have cell culture wells that are individually supplied with nutrients to allow independent challenge and parallel measurement of temporal responses under conditions that simulate interstitial flow.

Thus, the aim of this study was to develop a dynamic *in vitro* 3D model of the human airway epithelium that can be challenged apically while the basolateral secretion of mediators can be measured over time. Here we describe the design and fabrication of a multi-chamber culture device that integrates standard permeable filter supports containing fully differentiated PBECs with microfluidic flow to provide a constant basolateral supply of nutrients that simulates the circulation of fluids in the tissue. The microfluidic culture system was compatible with standard permeable filter inserts and was interfaced with a fraction collector for automated collection of the eluate to facilitate analysis of the kinetic response to an environmental challenge of differentiated PBECs at an air-liquid interface. The system was well tolerated by the differentiated cultures and, due to the small volume of the collected fractions, provided excellent sensitivity for mediator detection.

## Methods

### Design and fabrication of the microfluidic culture system

**Fig 1:**
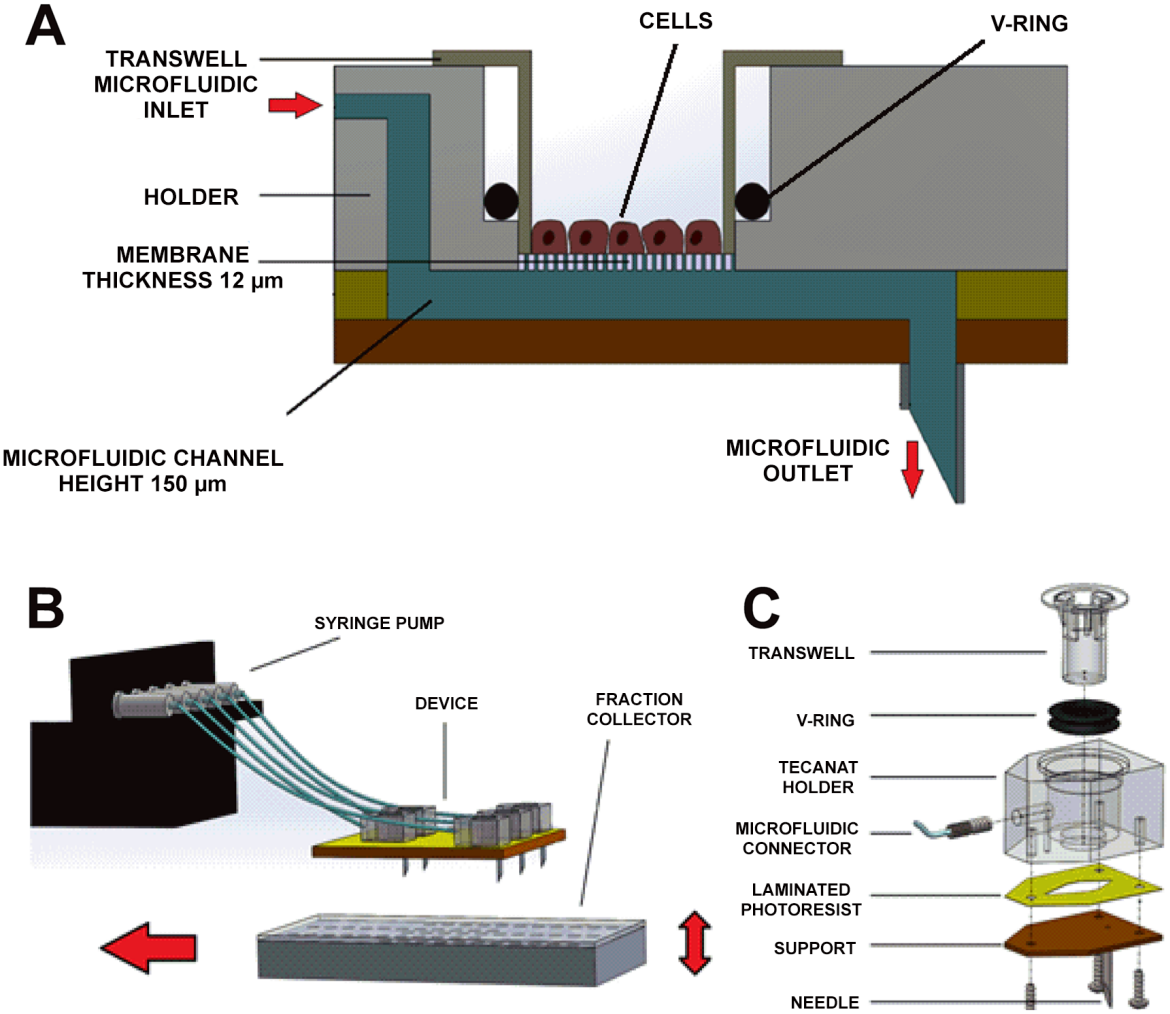
Design and assembly of the microfluidic culture system. a: Schematic section of a culture well: cells are cultured on a Transwell^®^ inserted in the holder. A V-ring is used to seal the system. b: Schematic of the setup: each well is connected to the syringe pump. A plate is placed under the device and is moved to allow for the time-resolved fraction collection. c: Exploded view of the assembly of a single well. Five layers of dry film photoresist are laminated on top of a support layer. A holder is screwed on top of the support layer and a Transwell^®^ is subsequently inserted together with the V-ring. The microfluidic inlet is screwed in the holder while the outlet needle is glued on the bottom of the support layer.

The microfluidic culture system consists of 5 separate chambers that each houses a conventional Transwell^®^, as shown in Fig 1. The design and fabrication is described in the online supplement (S1 Methods).

### Cell culture

Human PBECs were obtained by epithelial brushing using fiberoptic bronchoscopy from subjects selected from a volunteer database without any chronic airway diseases. All procedures were approved by the Southampton and South West Hampshire Research Ethics Committee and were undertaken following written informed consent. Culture of PBECs is described in the online supplement (S2 Methods). Filter supports with fully differentiated PBECs were inserted into the microfluidic culture system and perfused with basal medium at a flow rate of 30μL/hr. As a static culture control, filter supports with fully differentiated PBECs were cultured in standard cell culture wells containing 500 μL of basal medium. After a 1hr equilibration phase, differentiated cells were apically stimulated with grass pollen extract (67μL) of an equivalent of 1mg Timothy grass (*Phleum pratense*) or BEBM as control for 24hrs as previously described [16].

### Analysis of epithelial barrier responses

In the microfluidic culture system the basolateral flow was collected at 2hr intervals with the automated fraction collector. In static control experiments basolateral supernatant was collected at matching time points. The concentration of CXCL8/IL–8 in supernatants was analysed by Human CXCL8/IL–8 DuoSet ELISA kit (R&D Systems, Abingdon, UK). The physical barrier properties before and after stimulation were monitored by measuring TER.

### Immunofluorescence staining

After fixation with 4% paraformaldehyde, cells were permeabilised with 0.1% Triton X–100, blocked with 1% BSA in PBS and actin filaments were stained with Acti-Stain555-phalloidin (Cytoskeleton, Denver, Colorado, US) and AlexaFluor^®^488-conjugated mouse anti-human occludin antibody (clone OC-3F10, Life technologies) over night at 4°C. Subsequently, cells were washed extensively and mounted on slides using ProLong Gold antifade reagent with DAPI (Life technologies). Z-stacks were taken using LSM 6000 microscope (Leica Microsystems, Wetzlar, Germany). After deconvolution using Leica Application Suite software z-projections were performed using ImageJ software.

## Results

### Flow rate optimization

**Fig 2:**
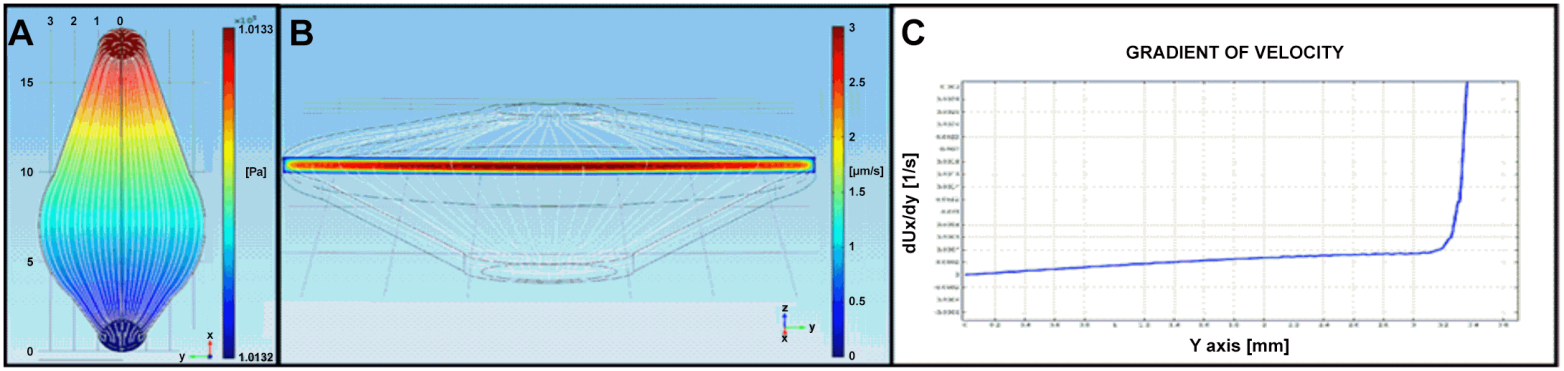
Simulation of the microfluidic flow in the chamber. a: Top view of the results of the simulation of half channel. Stream lines are represented in grey. The colours and the scale bar are relative to the pressure along the channel [Pa]. b: 3D view of the simulated system. Stream lines are represented in grey. A heat map of the values of the x component of the velocity in the widest section of the channel is reported together with the colour scale bar [μm/s]. c: Plot of the gradient of the x component of the velocity along the y-axis versus the distance from the centre of the channel.

**Fig 3:**
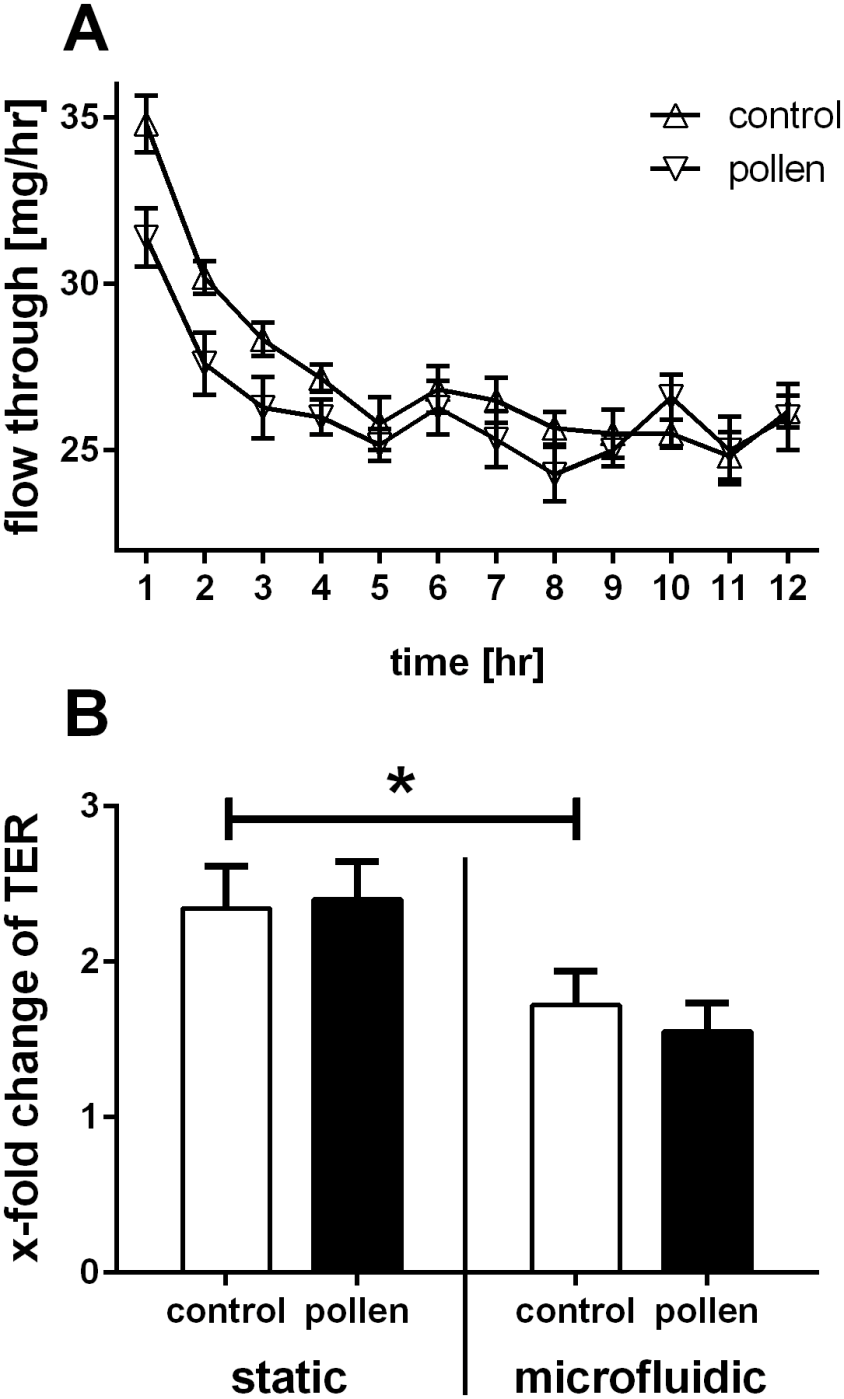
Characterization of microfluidic culture system. Differentiated PBECs were cultured in the microfluidic culture system and after an equilibration phase of 1h apically exposed to pollen extract. a: The flow rate of pollen-challenged and control wells was determined by measuring the outlet volume per hour for a period of 12h. n = 7 independent experiments with different donors. b: The transepithelial electrical resistance (TER) of differentiated PBECs was measured before and after microfluidic culture conditions without or with pollen challenge. Cells cultured in common culture conditions without flow were used as static controls. TER was normalized to the respective value before the start of the experiment. n = 15 independent experiments using 13 different donors; *: p≤0.05.

**Fig 4:**
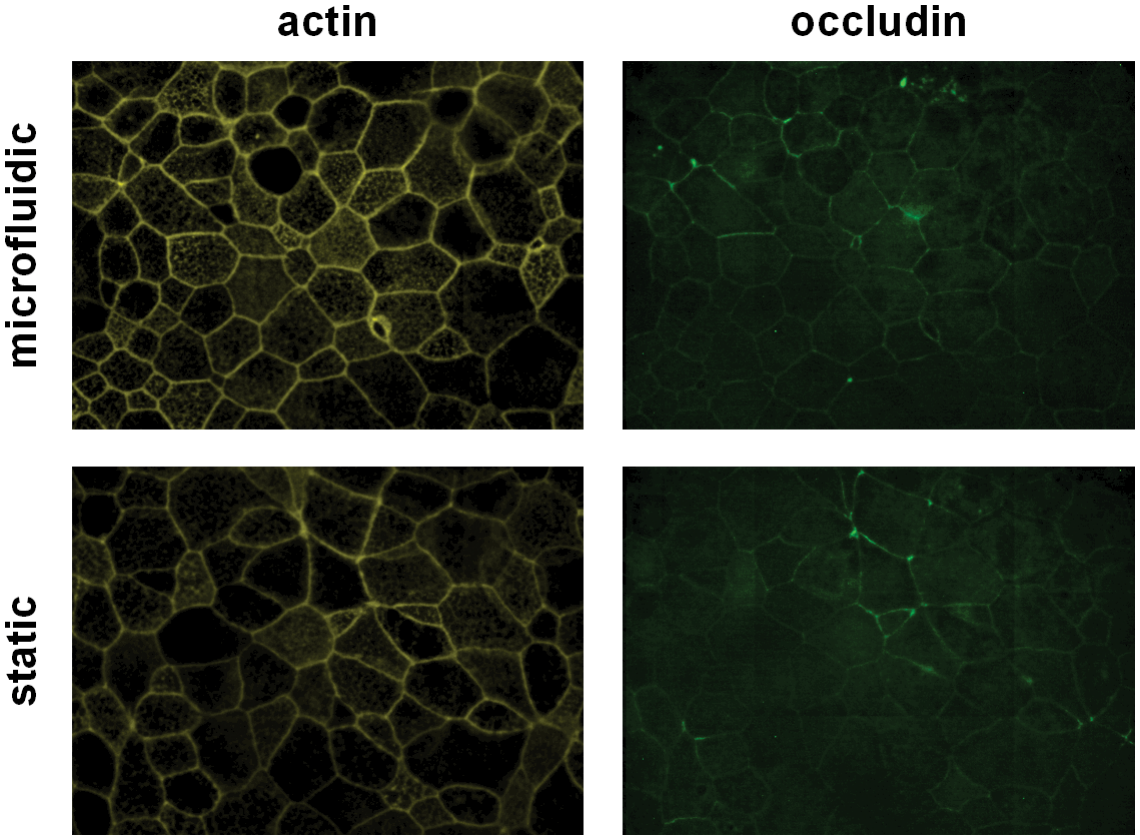
Cell viability and barrier integrity of differentiated PBECs in microfluidic compared to static culture conditions. After 24h in microfluidic or static culture conditions, actin filaments (yellow) and the tight junction protein occludin (green) were stained by immunofluorescence. Images are representative of 3 independent experiments using 3 different donors.

A flow rate of 30μL/hr corresponds to a velocity of around 3μm/s in the widest part of the microfluidic chamber underneath the Transwell^®^ support (Fig 2). By collecting fractions from the device each hr, we were able to assess the stability of the system. As shown in Fig 3a, the volumetric flow rates stabilised after 4hrs to a volume of approximately 27μL/hr. The higher volumes seen over the first 3hrs can be explained by a clearance of the apical liquid (added for stimulation by the cells) by absorption across the epithelial cell layer, a process that can be regulated by active ion transport across the epithelium leading to iso-osmolar fluid clearance [17]. After 24hrs, the apical liquid volume in the microfluidic culture system was approximately half of that in static culture conditions and closely resembled an air-liquid interface culture. Apical stimulation of differentiated PBECs with pollen extract had no significant effect on the basolateral volume, indicating an intact physical barrier without additional loss of fluid from the apical compartment. The barrier integrity was evaluated by measuring the ionic permeability across the membrane by TER. After stimulation, the TER increased under either static or microfluidic culture conditions (Fig 3b). This increase in TER is due to the application of liquid to the apical surface [18, 19]. However, the fold increase in TER in microfluidic culture was significantly lower than under static culture conditions. This is probably due to the reduction in the apical fluid volume as a result of enhanced fluid clearance under microfluidic culture conditions. Importantly, differentiated PBECs in microfluidic culture did not show an altered response to pollen extract compared to static culture conditions as measured by TER. Since the TER is a good indicator of the integrity of the physical barrier, differentiation status and viability of the epithelium, it is highly unlikely that microfluidic culture conditions cause a change of these properties. The integrity of the epithelial barrier during microfluidic culture conditions is further shown by immunofluorescence staining with Acti-Stain555-phalloidin (Fig 4), which showed that the actin filaments of the cytoskeleton were similar under microfluidic and static culture conditions in their cellular localisation including stress-induced filaments. Furthermore, the transmembrane tight junction protein occludin, which is a regulator of tight junction sealing and sensitive to barrier pertubations ([20–22]ref) was localised at cell-cell junctions and no difference in the localisation was observed between microfluidic and static culture conditions confirming the integrity of the physical barrier.

### Release of the inflammatory mediator IL–8

**Fig 5:**
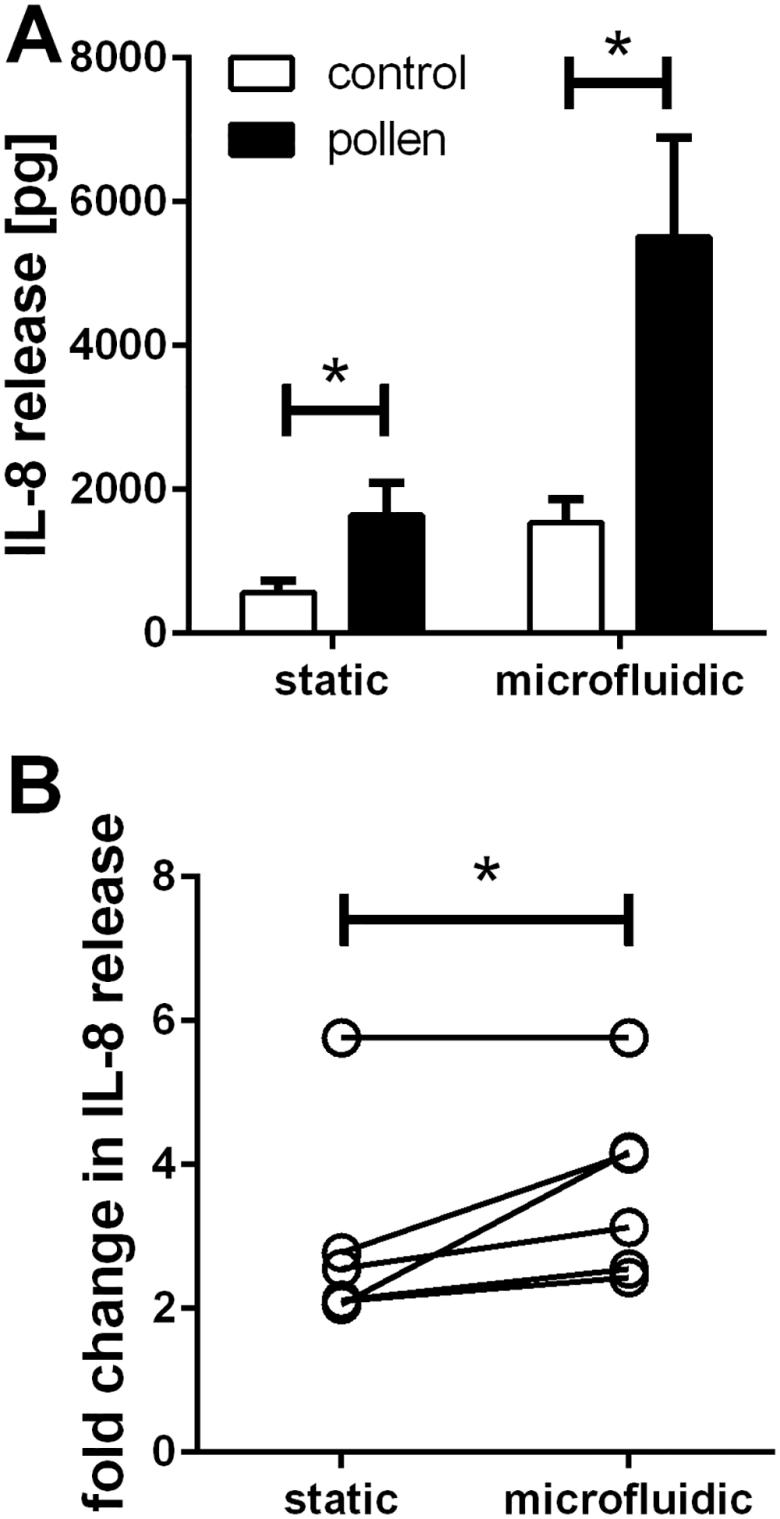
Release of IL–8 in microfluidic compared to static culture conditions. Basolateral release of IL–8 by differentiated PBECs after challenge with pollen was analysed by ELISA. n = 6 independent experiments using 5 different donors. a: Overall amount of IL–8 released by differentiated PBECs in a period of 24h after pollen treatment. b: Comparison of pollen induced IL–8 release in static and microfluidic culture condition. The x-fold change in pollen induced IL–8 release compared to untreated control is shown. Linked data points represent experiments run in static and microfluidic culture conditions in parallel with matching PBEC donor. *: p≤0.05 (Wilcoxon).

After stimulation with pollen extract, differentiated PBECs released the inflammatory chemokine CXCL8/IL–8 into the basolateral compartment (Fig 5). In static as well as microfluidic culture conditions, pollen induced a significant increase in IL–8 release (Fig 5a). The overall amount of IL–8 released basolaterally over a 24hrs period was significantly increased in microfluidic compared to static culture conditions. Additionally, the fold increase in IL–8 release induced by pollen in microfluidic culture conditions was significantly higher than in static conditions (Fig 5b). This increase in overall production of IL–8 in microfluidic culture conditions might be explained by reduced feedback inhibition, since released IL–8 is constantly removed via the microfluidic flow. In static culture conditions IL–8 accumulates constantly over time and may inhibit production by a negative feedback loop. To test this hypothesis further experiments investigating the transcriptional and translational control of CXCL8/IL–8 expression are required.

### Time-dependent release of IL–8

**Fig 6:**
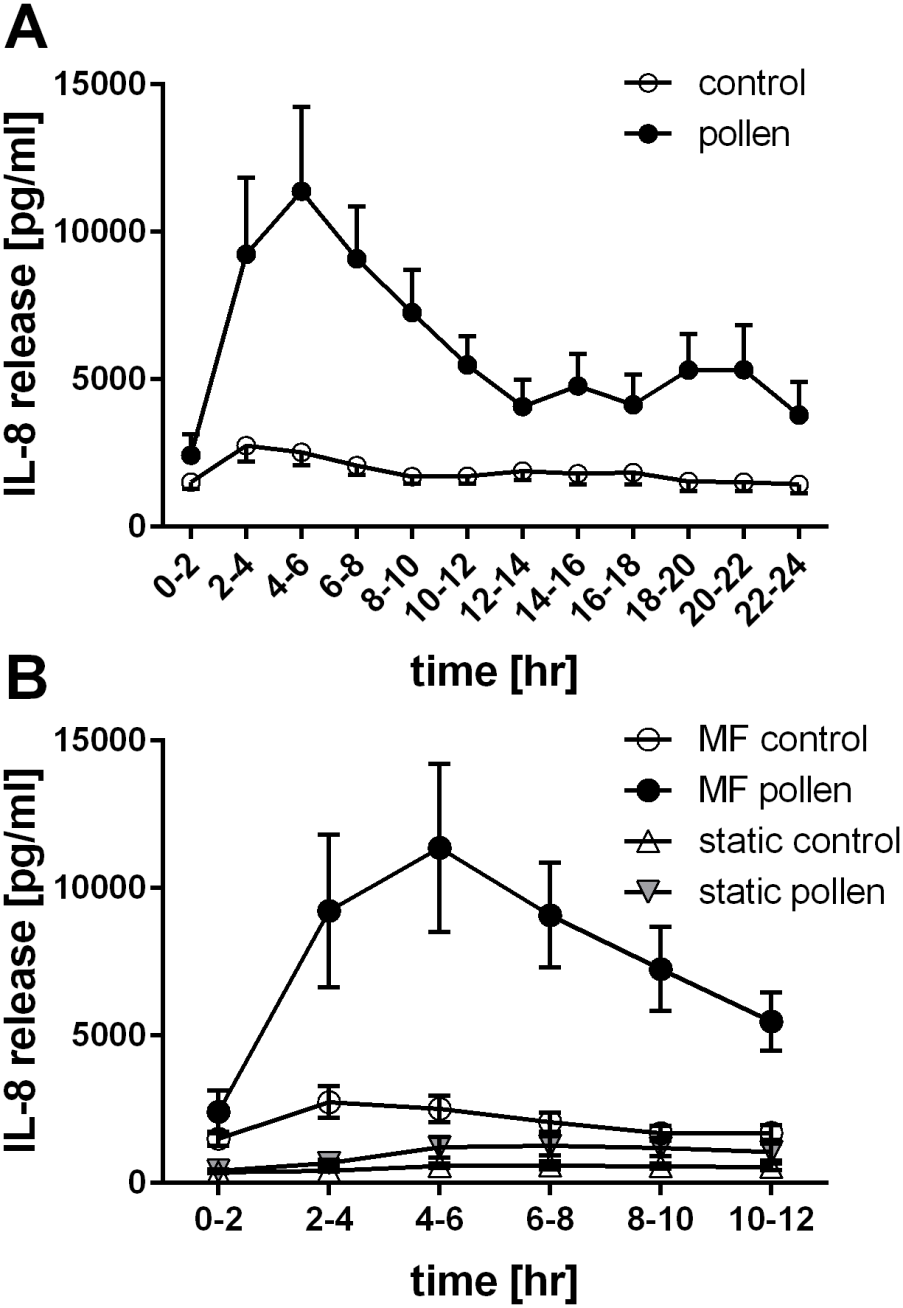
Time-dependent release of IL–8 by differentiated PBECs induced by pollen. a: Using the microfluidic culture system, basolateral flow was collected for 2h periods over 24h. Release of IL–8 was analysed by ELISA. n = 15 independent experiments using 13 different donors. b: Comparison of pollen induced IL–8 release in static and microfluidic culture conditions in 2h intervals over a 12h period. In static culture conditions, aliquots of basolateral medium were taken at matching time points. Release of IL–8 was analysed by ELISA. Microfluidic culture (MF) n = 15; static n = 5 independent experiments using 13 and 4 different donors respectively.

Microfluidic culture conditions coupled with the automated fraction collector provided the capability to analyse time-dependent release of IL–8 over a period of 24hrs. After stimulation with pollen extract, differentiated PBECs rapidly released significant amounts of IL–8 (Fig 6a), reaching a maximum at 6hrs after stimulation. The release of IL–8 thereafter reduced up to 14hrs after stimulation, stabilizing at a constant value of around 5ng/mL. A slight shoulder between 20–22hrs is seen, indicative of a second peak in IL–8 release; however, this increase was not significant. Interestingly, such a time-dependent release of IL–8 could not be detected in static culture conditions (Fig 6b), where aliquots of basolateral medium at matching time points were analysed. Due to the higher culture medium volume, the concentration of IL–8 released in the basolateral medium of the static cultures was much lower than in the microfluidic culture system, which made it more difficult to measure the released mediator. Furthermore, due to the accumulation of IL–8 over time in the static culture, it was not possible to detect the kinetic profile of the release of IL–8. In summary, the microfluidic culture conditions enabled analysis of time-dependent mediator release by differentiated PBECs in response to environmental impacts with a much higher sensitivity than conventional static culture conditions.

## Discussion

We have developed a novel microfluidic culture system which allows experimental challenge of up to 5 parallel cultures of differentiated PBECs contained in standard permeable filter supports combined with integration of an automated fraction collector to allow high resolution temporal analysis of mediator release. This system allows use of conventional culture techniques in which PBECs can be differentiated on permeable supports to produce a 3D construct of the airway epithelium and, once fully differentiated, the cultures can be transferred to the microfluidic culture system. In the dynamic culture model presented here, the microfluidic flow guarantees a continuous supply of nutrients and removes metabolites to more accurately reflect the circulation of fluids *in vivo*. It has been shown that the interstitial flow in the tissue has a velocity between 0.1–2.0μm/s and can reach up to 4.0μm/s [23, 24]. In our microfluidic culture system the velocity of the flow was maintained in this range to reflect the *in vivo* situation. Under these conditions, airway epithelial polarity was maintained in the microfluidic system as determined by TER, indicating a differentiated and viable airway epithelium. Combining the device with an automated fraction collector provided a means of analysing, in-depth, the kinetics of mediator release induced by environmental challenge. Using the system, we identified that apical exposure to grass pollen caused an early basolateral inflammatory response, inducing release of IL–8 from differentiated PBECs within the first 6hrs. To our knowledge, this is the first report showing the detailed kinetics of mediator release by differentiated PBECs induced by an environmental agent.

A detailed understanding of the kinetics of airway epithelial mediator release in response to environmental impacts should significantly enhance our knowledge of environment-to-epithelial-to-tissue signalling pathways that are involved in airway homeostasis. Our data suggest that microfluidic culture conditions offer the potential for high resolution temporal profiling of epithelial barrier responses under conditions that more closely mimic the *in vivo* situation. Analysing the kinetic release of inflammatory mediators by airway epithelial cells should provide insights into the temporal primacy of certain mediators over other cytokines that regulate inflammation. Such data is crucial for targeting appropriate anti-inflammatory drugs. Furthermore, by enabling use of differentiated PBECs originating from individuals with chronic lung diseases, this dynamic 3D model of the airway mucosa has great potential to enhance our understanding of underlying disease mechanisms, facilitate identification of new therapeutic targets and optimize existing therapies.

Over the last 5 years, considerable effort has been invested into tissue engineering and design of microfluidic culture platforms for epithelial cells from different origins including gut, liver, skin, kidney and lung [25, 26]. Most microfluidic platforms for studying epithelial cell behaviour incorporate a permeable membrane that divides the system into apical and basolateral compartments. Epithelial cells are generally grown on the apical side of the permeable membrane. Many of these models are designed to address specific aspects of lung biology. For example, a microfluidic platform was developed to analyse the dynamics and mechanical stresses caused by liquid plugs in small airways using primary human cells [11, 27, 28]. Using the alveolar epithelial cell line A549, Nalayanda *et al*. (2009) [29] developed an alternative model with comparable larger basolateral channel volume. However, a specific biological question was not addressed with this model. A revised and modified version was later developed in order to analyse ventilator-induced injury of the respiratory epithelium using cell lines as a model [30]. In this platform up to 4 cell culture wells were interconnected with media and airflow channels, however this did not allow for individual treatment and analysis. Another interesting model of the alveolar epithelial barrier was developed by Ingber and colleagues [9] incorporating cyclic mechanical strain of breathing. A co-culture model of the alveolar epithelium using the cell line A549 and endothelial cells under cyclic strain was used to analyse the barrier modulating effect of nanoparticles and neutrophil adhesion. However, the suitability of this platform for primary human alveolar epithelial cells still needs to be confirmed. A similar approach to our microfluidic culture system has been used by Wang *et al*. (2014) [14] for the culture of human nasal epithelial cells. Here, the aim was to monitor cilia function with high resolution microscopy. Due to the limited optical working distance of high magnification lenses, they used an ‘upside-down’ approach with cells on the basolateral side of a permeable filter support, directly facing a microfluidic channel. Airflow and medium were supplied from the top of the insert without continuous flow. This model was used to analyse cilia beat frequency in response to gaseous substances like formaldehyde. The combined use of primary cells and microfluidics has recently highlighted the dependence of the transport behaviour of kidney cells on external shear stresses and flow conditions [31]. However, none of the microfluidic culture models described in the literature are suitable for high resolution analysis of the mediator responses of differentiated PBECs to environmental stimuli.

Membrane based microfluidic platforms are also a useful tool for studying toxicology [32] and the permeability of drugs across epithelial surfaces. Using the Caco–2 cell line as a model of the intestine epithelium, Gao *et al*. (2013) [33] developed an integrated microfluidic device with high resolution mass spectrometry in order to detect the passage of curcumin across the epithelial barrier. A similar approach could be transferred into the microfluidic culture system described here using primary human airway epithelial cells differentiated on commercially available permeable filter supports at the air interface and used for analysing the passage of respiratory drugs across the airway epithelium or even the passage of environmental airborne substances and fumes.

The advantage of our microfluidic platform is that PBECs can be differentiated separately using conventional well established culture techniques, which allows the user to regularly check the cells over the 3 week differentiation period, e.g. for differentiation status or contaminating infections. This is particularly important when using PBECs from individuals with chronic lung diseases as they are associated with defects in their epithelial barrier properties and commonly bacterial infections, which increase the contamination risk to cultures. Since the microfluidic culture system presented here is based around commercially available permeable filter supports, which are widely used and tested in established cell culture models, this microfluidic platform can easily be translated into other epithelial models, like the skin, gut, liver or kidney. Our microfluidic system combines 5 separate wells into one device for parallel testing, and this could easily be scaled for higher throughput. It is user-friendly and suitable for any cell-culture trained individual without a specialized microfluidic background. Furthermore, the model could also be used for drug safety and permeability studies with provision of additional kinetic information. By using commercial available cell culture inserts and already established cell culture techniques our microfluidic culture system can easily be expanded to more complex co-culture systems, e.g. combining airway epithelial cells with mesenchymal or endothelial cells recapitulating the cellular composition of the airway tissue *in vivo*.

In summary, we have developed a microfluidic culture system for primary epithelial cells which allows the kinetic analysis of barrier responses to environmental impacts and with a much higher sensitivity than conventional static culture systems. The dynamic nature of the system allows the continuous exchange of fluids and mediators which more closely mimics the interstitial flow *in vivo*. This dynamic culture system will be a useful tool for analysing the mechanisms of chronic lung diseases and help identifying new therapeutic strategies, as has potential for toxicological or pharmacological studies as well.

## Supporting Information

S1 Methods Microfluidic culture system design and fabrication. (DOCX)

S2 Methods Epithelial cell culture. (DOCX)
