# Supplementary material for: Temporal Monitoring of Differentiated Human Airway Epithelial Cells Using Microfluidics
Source: PLoS One. 2015 Oct 5;10(10):e0139872. doi: 10.1371/journal.pone.0139872 (PMC4593539; doi:10.1371/journal.pone.0139872)
Supplement: S1 Methods — (DOCX) [file pone.0139872.s001.docx]

**Online Supplementary**

**Temporal monitoring of differentiated human airway epithelial cells using microfluidics**

Cornelia Blume^1*^, Riccardo Reale^2^, Marie Held^2^, Timothy M. Millar^1^, Jane E. Collins^1^, Donna E. Davies^1,3,4^, Hywel Morgan^2,3^, Emily J. Swindle^1,3,4^

^1^Academic Unit of Clinical and Experimental Sciences, Faculty of Medicine, University of Southampton, Southampton, United Kingdom

^2^Electronics and Computer Sciences, Faculty of Physical Sciences and Engineering, University of Southampton, Southampton, United Kingdom

^3^Institute for Life Sciences, University of Southampton, Southampton, United Kingdom

^4^National Institute for Health Research, Respiratory Biomedical Research Unit, University Hospital Southampton, Southampton, United Kingdom.

*****Corresponding author

Email: [c.blume@soton.ac.uk](mailto:c.blume@soton.ac.uk) (CB)

**S1_Methods**

*Design and fabrication of the microfluidic culture system.* Transwells^®^ are commercially available plastic supports with 6.5mm diameter permeable polyester membrane (pore size 0.4 µm) bottoms on which cells are cultured. The Transwell^®^ holders were made from Tecanat (Ensinger, Waterlooville, UK). The device was designed to provide a constant volumetric flow beneath the Transwell^®^ membrane enabling continuous perfusion of the cells. Fluid flow was achieved beneath the Tecanat holder through a channel (width 7 mm, length 18 mm) made by laminating five layers of dry film photoresist Ordyl SY 355 (30 µm thick, Elga Europe, Italy) to the top of the supporting substrate. The dry film resist was exposed to UV light and developed to define the microchannels resulting in a total channel depth of 143.4 μm ± 2.7. The Tecanat holder was then bonded to the laminate stack with silicone glue (RS Components Ltd, Corby, UK) and secured in place using screws. Transwells^®^ were inserted into the Tecanat holder and held in place with silicone V-rings (Sealforce, Dundee, UK). After sterilisation with 70% ethanol and thorough washing, the complete system was located in a humidified incubator at 37°C and 5% CO_2_. Each separate Transwell-chamber was connected to a syringe and the flow was controlled with a single 10-syringe pump (Harvard Apparatus, Kent, UK); the fluid from underneath the Transwell^®^ was collected using a custom designed fraction collector.

The fraction collector was designed to interface with the microfluidic devices and to automatically collect samples from each of the five Transwell^®^-chambers at a frequency of typically once per hour or every 2 hrs. The collector held a rack of 0.2 mL microtubes, each of which were covered with a 2 mm thick poly(dimethylsiloxane) (PDMS) membrane to prevent evaporation of the samples. To collect samples, the fraction collector pushes a set of 5 tubes up against the needles coming from the Transwell^®^ chambers, piercing the membrane and allowing the culture eluate to be collected. After 1 or 2 hours, the tubes were withdrawn and the next set of 5 tubes was interfaced with the device.

*Optimisation of the flow channel design*. In designing the flow chamber beneath the Transwells^®^ it was important to maintain a very low pressure difference (to avoid leakage through the cell layer) and to have a uniform flow velocity across the channel width. A finite element model of the microchannel was built in Comsol Multiphysics 3.4 (Comsol Inc., Sweden), and an example of a simulation is shown in Fig. 2. At the flow rate used in these experiments (30 µL/hr) the backpressure on the membrane was negligible (ΔP< 1 Pa). The Reynolds number was very low (Re~10^-3^) giving a uniform velocity profile beneath the membrane.
