# Supplementary material for: Temporal Monitoring of Differentiated Human Airway Epithelial Cells Using Microfluidics
Source: PLoS One. 2015 Oct 5;10(10):e0139872. doi: 10.1371/journal.pone.0139872 (PMC4593539; doi:10.1371/journal.pone.0139872)
Supplement: S2 Methods — (DOCX) [file pone.0139872.s002.docx]

**Online Supplementary**

**Temporal monitoring of differentiated human airway epithelial cells using microfluidics**

Cornelia Blume^1*^, Riccardo Reale^2^, Marie Held^2^, Timothy M. Millar^1^, Jane E. Collins^1^, Donna E. Davies^1,3,4^, Hywel Morgan^2,3^, Emily J. Swindle^1,3,4^

^1^Academic Unit of Clinical and Experimental Sciences, Faculty of Medicine, University of Southampton, Southampton, United Kingdom

^2^Electronics and Computer Sciences, Faculty of Physical Sciences and Engineering, University of Southampton, Southampton, United Kingdom

^3^Institute for Life Sciences, University of Southampton, Southampton, United Kingdom

^4^National Institute for Health Research, Respiratory Biomedical Research Unit, University Hospital Southampton, Southampton, United Kingdom.

*****Corresponding author

Email: [c.blume@soton.ac.uk](mailto:c.blume@soton.ac.uk) (CB)

**S2_Methods**

*Cell culture*. PBECs were cultured in bronchial epithelial growth medium (BEGM; Lonza, Basel, Switzerland) and differentiated at the air-liquid interface (ALI) for 21 days on Transwell^®^ permeable supports (6.5 mm diameter, polyester membrane, pore size 0.4 µm, Corning Life Sciences, Amsterdam, The Netherlands) as previously described [E1]. The differentiation status of the cultures was monitored by measuring the physical barrier properties by trans-epithelial resistance (TER) every 7 days using chopstick electrodes and an EVOM Volt-Ohm meter (World Precision Instruments, Aston, UK). After 21 days, fully differentiated PBECs with a TER above 1000Ω were starved 24h before stimulation with bronchial epithelial basal medium (BEBM; Lonza) supplemented with 1.5 µg/mL BSA (Sigma, Poole, UK), 1x Insulin-Transferrin-Selenium and 50 U/mL penicillin, 50 µg/mL streptomycin (Life technologies, Paisley, UK).

**References**

E1 Xiao C, Puddicombe SM, Field S, Haywood J, Broughton-Head V, Puxeddu I, et al. Defective epithelial barrier function in asthma. *J Allergy Clin Immunol* 2011; 128:549-56 e1-12.
